# Maternofetal outcome of asymptomatic bacteriuria among pregnant women in a Nigerian Teaching Hospital

**DOI:** 10.11604/pamj.2017.27.69.10492

**Published:** 2017-05-30

**Authors:** Kenneth Ebele Izuchukwu, Emmanuel Okwudili Oranu, Goddy Bassey, Ngozi Clare Orazulike

**Affiliations:** 1 Department of Obstetrics and Gynaecology, University of Port Harcourt Teaching Hospital, Port Harcourt, Rivers State, Nigeria

**Keywords:** Asymptomatic bacteriuria, prevalence, fetomaternal outcome, Port Harcourt

## Abstract

**Introduction:**

Asymptomatic bacteriuria has been reported to be associated with adverse pregnancy outcome. This study sought to determine the prevalence and complications of asymptomatic bacteriuria amongst parturient in the University of Port Harcourt Teaching Hospital (UPTH).

**Methods:**

The study was a prospective cohort study involving 220 eligible antenatal attendees. Urine culture and sensitivity was conducted for each participant and the fetomaternal outcome between affected and unaffected women were compared and p value <0.05 was considered significant.

**Results:**

Sixty-five of the participants had asymptomatic bacteriuria giving a prevalence of 29.5%. Twenty-three (35.4%) cultures yielded *Klebsiella spp* while Fifty-eight (89%) of the cultured organisms were sensitive to Nitrofurantoin. There was no statistical difference in the rate of prelabour rupture of membranes, preeclampsia, preterm delivery, birth asphyxia and low birth weight between affected and unaffected women.

**Conclusion:**

Contrary to widely held view, there was no significant increase in adverse pregnancy outcome amongst affected women.

## Introduction

Asymptomatic bacteriuria (AB) is defined as the presence of actively multiplying bacteria in the urinary tract excluding the distal urethra in a patient without any obvious urinary symptom [1,2]. The presence of 100,000 or more colony forming units of a single bacteriuria per milliliter of two consecutive clean catch urine specimens or a single catheter specimen in absence of urinary symptoms and signs has been taken as significant in making the diagnosis of asymptomatic bacteriuria [1–3]. Asymptomatic bacteriuria can be seen in general population but occurs more in pregnancy due to physiological changes that occur in pregnancy [1,2]. These changes include increased level of hormones principally serum progesterone which causes relaxation of the smooth muscles of the urinary tract, increased alkalinisation of urine by increased excretion of bicarbonates and mechanical compression of the ureters by the enlarging uterus [3]. These changes enhance colonisation of the urinary tract by organisms such as Escherichia coli (E.coli), Klebsiella, Proteus and Staphylococcus species. Most of the women whose urine are colonized are asymptomatic and hence never receive treatment, while few go on to develop frank symptoms and signs of urinary tract infection [3]. The reason for the asymptomatic clinical state has been related to the absence of Type 1 fimbriae found in certain strains of bacteria particularly E. coli [4]. This fimbriae is immunogenic and its presence initiates the immune/inflammatory response, which leads to development of symptoms whereas its absence leads to absence of symptoms [4]. In making the diagnosis of asymptomatic bacteriuria, technically, two consecutive samples are ideally collected. This helps to reduce the incidence of false positive results. The use of midstream urine culture has been shown to be superior to the other methods of screening as it equally studies the antimicrobial sensitivity [5–8]. Quiroqa-Feuchter et al in their study, collected samples monthly from the patients and reported a high prevalence of 25% among their study population [9]. Okonkwo and co workers in Benin, Nigeria reported the use of chlorhexidine in diagnosing asymptomatic bacteriuria with 100% sensitivity, but it’s low accuracy of 40% and specificity of 28.5% makes this method of screening unreliable [10]. The burden of bacteriuria appears to be the same in asymptomatic and established cases of urinary tract infection as both have been associated with increased incidences of preterm labour, anaemia, pre-eclampsia, prelabour rupture of membranes and puerperal sepsis [2,11]. Despite association of asymptomatic bacteriuria with these adverse pregnancy outcomes, screening and treatment is not pursued with much vigor as is done for frank urinary tract infection. This pioneer study in the University of Port Harcourt Teaching Hospital (UPTH) sought to establish the prevalence and any adverse effect of asymptomatic bacteriuria.

## Methods

This study was a prospective cohort study of 220 pregnant women presenting for antenatal care at the UPTH. Ethical approval for the conduct of this study was obtained from the Ethics committee of the hospital. A cross sectional survey was done to determine the prevalence of asymptomatic bacteriuria in pregnancy at booking using two consecutive clean catch midstream urine specimens from participants. The urine samples were collected at consecutive times of voiding. The socio-demographic characteristics of the participants and other relevant information were recorded in a structured proforma. For all positive cases, the prevailing organisms were recorded and the sensitivity pattern to antibiotics determined. The women that tested positive had a repeat midstream culture done four weeks later or at 28 weeks and at 36 weeks of gestation. For each of the positive case, a similar participant in terms of maternal age, parity and gestational age was selected from the negative group as control and fetomaternal outcome between positive and negative cases were compared. Similarly, the urine samples of the negative group were repeated four weeks later or at 28 weeks and at 36 weeks of gestation. The two groups were closely monitored in the course of pregnancy up to delivery and immediate puerperium. The women who were recruited for the study were counselled on symptoms of urinary tract infection such as dysuria, frequency, urgency, suprapubic and loin pains and instructed to report to the researcher by telephone on development of these symptoms. The women who developed symptoms and signs of urinary tract infection were treated. Similarly, the women who were previously negative but developed bacteriuria in subsequent urine tests, or frank urinary tract infection were treated and excluded from the study. All the women who participated in the study were followed up till delivery and cases of anaemia, preterm labour, pre-labour rupture of membranes were documented. The gestational age at delivery, birth weight and Apgar scores were also documented. Statistical analysis of generated data was done using SPSS soft ware version 19 (Armonk, NY:IBM Corp 2010) and comparison of generated data between positive and negative cases was done using Chi square test and student ‘t’ test and P value < 0.05 was considered as significant.

**Determination of sample size:** The sample size was calculated from the formula [12] n= Z^2^P(1-P)/d^2^ using a prevalence of AB of 15% as reported by Ezeome et al in 2006 [5], tolerance error of 5% and an attrition rate of 10%. The allowed minimum sample size for this study was 215. Therefore 220 women were recruited for this study. Fifty women were recruited weekly and the desired sample size was recruited over a 5-week period. The participants were followed up over 5-7 months. The study was conducted between April and December, 2013.

**Inclusion criteria:** Women who gave consent to participate in the study, were certain of their last menstrual period, and did not have the medical conditions listed below under exclusion criteria were eligible to take part in the study.

**Exclusion criteria:** Women who did not consent to take part in the study, those who were not certain of their Last Menstrual Period (LMP) and did not have an early ultrasound scan to date the pregnancy were excluded from the study. Also excluded were women with multiple gestation, symptoms of urinary tract infection, Human immuno-deficiency virus (HIV), sickle cell anaemia, diabetes mellitus and those who delivered outside UPTH despite being part of the study.

**Sample processing:** Each patient recruited was allotted an identification number. The midstream urine samples collected were correctly labelled with the participant’s identification number. About 10 milliliters of urine was collected from each participant. The urine samples collected were sent to the Microbiology Laboratory where a senior microbiologist supervised by a consultant medical microbiologist carried out processing and analysis. Urine microscopy, culture and sensitivity were conducted using the Kirdy Buer technique [13] and the results were documented in each participant’s proforma. A diagnosis of significant bacteriuria was made when there were at least 10^5^ colony forming unit of a single bacteria per milliliter of urine inoculated on chocolate and blood agar plates. Subsequent culture in cystein lactose electrolyte deficient agar (CLED) was carried out to determine the specific organism as well as antibiotic sensitivity.

## Results

Two hundred and twenty women were recruited for the study. Sixty-five of them had asymptomatic bacteriuria giving a prevalence of 29.5%. Sixty-five women who were negative for bacteriuria were cross-matched as control. The mean ages of the positive and negative groups were 30 ± 4.5 and 29.8 ± 4.3 years respectively. The mean parity of the women was 2.2 ± 0.8 and 2.2 ± 0.7 for the study group and control respectively. The mean gestational age at booking was 23 ± 2.1 weeks for the entire study population. All the women recruited for the study were married. Using the Olusanya et al [14] classification 8 of the women with bacteriuria were of upper socioeconomic class, 42 (64.64%) were of middle socioeconomic class while 15 (23.07%) were of the lower socioeconomic class. **Table 1** shows the socio-demographic characteristics of women with AB and revealed that most women with AB were aged 30-34 years and were Para 1. Out of the 65 positive urine culture, 23(35.38%) grew Klebsiella, 16 (24.61%) grew Escherichia coli, 13(20.00%) grew Staphylococcus, 7(10.77%) grew Pseudomonas and 6(9.23%) grew Coliform organisms. Fifty-eight (89.23%) of the organisms were sensitive to Nitrofurantoin 50 (76.92%) were sensitive to Ceftaxidime, 42 (64.61%) to Gentamycin, 40 (61.54%) to Augmentin and Ofloxacin respectively and 38 (58.46%) to Cefuroxime. Seven women (10.7%) in the affected group subsequently developed urinary tract infection (UTI) while two (3.1%) from the unaffected group developed UTI and the difference was not statistically significant (p=0.0821, Odds ratio (OR) =3.8). However, women with AB were about four times more likely to develop UTI. The distribution of organisms causing UTI were similar to that of AB with Klebsiella being the most prevalent in 6(66.7%) out of the nine women who had UTI. These women with UTI were treated and excluded from the study while 5 women in the affected group were lost to follow up. Therefore the fetomaternal outcome of asymptomatic bacteriuria amongst the study population was determined in 53 affected women and 53 unaffected women who were used as matched controls. The mean Packed Cell Volume (PCV) were 31.5±2.9 and 31.9±3.4 for the affected and the unaffected women respectively and the difference was not statistically significant (P=0.963). Among the affected women, 17 (32.1%) had anaemia (PCV of less than 30%) While 12 (22.6%) of the women without AB had anaemia and the difference was not statistically significant (x2 = 1.19, P = 0.2759, OR=1.61). There were three cases (5.7%) of pregnancy-induced hypertension (PIH) amongst the affected women while one case (1.9%) of PIH occurred amongst the unaffected women and the difference was not statistically significant (P = 0.308, OR=3.12). However, women with AB were three times more likely to develop PIH. There was no case of pre-labour rupture of membranes and preterm labour in both the affected and unaffected women.

**Table 2** Shows pregnancy complications between affected and unaffected women and revealed no significant difference in complications between women with AB and those without AB. The mean gestational age at delivery was 38.6±1.8 and 39±1.4 for affected women and unaffected women respectively and the difference was not statistically significant (p=0.161). The mean birth weight of the babies were 3.3kg ± 0.48 and 3.5kg ± 0.0.4 for affected and unaffected women respectively. There was no statistical difference (p=0.271). There was no case of preterm delivery or low birth weight in affected and unaffected mothers. The mean Apgar score at the first and fifth minute was 7.8 and 8.9 respectively in the affected group and 8.1 and 9.0 respectively for the unaffected women. There was no significant difference in the first and fifth mean Apgar score between the affected and unaffected women, (P=0.431, P=0.648). No baby had birth asphyxia amongst the entire study population and none was admitted in the special care baby unit. Three women (5.7%) had puerperal sepsis following caesarean sections; two from the study group and one (1.9%) from the control group and the difference was not statistically significant (p =0.308, OR=3.12). However, women with AB were three times more likely to develop puerperal sepsis than those without AB. **Table 3** shows the pregnancy outcome between affected and unaffected women with no significant difference between both groups.

**Table 1:** Sociodemographic characteristics of women with AB

Age (years)	Frequency (65)	Percentage (%)
< 20	1	1.54
20-24	5	7.69
25-29	23	35.38
30-34	26	40.00
>35	10	15.38
Parity		
Para 0	13	20.00
Para 1	30	46.15
Para 2-4	21	32.30
Para 5 and above	1	1.54
**Educational level**		
Primary	17	26.15
Secondary	36	55.38
Tertiary	12	18.46
**Socioeconomic status**		
Low	15	23.07
Middle	42	64.61
High	8	12.30

**Table 2:** Pregnancy complications compared between affected and unaffected women

Complication	Positive for AB	Percentage	Negative for AB	Percentage	P-value
Anaemia	17	32.1	12	22.6	0.275
PIH	3	5.7	1	1.9	0.308
UTI	7	10.7	2	3.1	0.082
IUFD	1	1.9	0	0	0.5
Preterm labour	0	0	0	0	-
PROM	0	0	0	0	-

AB - Asymptomatic Bacteriuria PIH- Pregnancy Induced Hypertension UTI- Urinary Tract Infection IUFD - Intrauterine Fetal Death PROM - Pre-labour Rupture of Membrane

**Table 3:** Pregnancy outcome compared between affected and unaffected women

Pregnancy outcome	Positive for AB	Percentage	Negative for AB	Percentage	P- value
Perinatal death	1	1.9	0	0	0.5
Puerperal sepsis	3	5.7	1	1.9	0.308
LBW	0	0	0	0	-
Birth Asphyxia	0	0	0	0	-
Admitted in SCBU	0	0	0	0	-

AB - Asymptomatic Bacteriuria LBW - Low Birth Weight SCBU - Special Care Baby Unit

## Discussion

This study found a prevalence of 29.5% of AB among the antenatal population at UPTH. Earlier studies reported lower incidences of 4-25% [5,11,15–17]. Akerele et al in Benin reported a higher prevalence of 88% in 2001 [18]. This wide variation in prevalence has been attributed to varying socio-demographic characteristics of the population studied with higher incidences reported in developing countries. The highest prevalence was found in women with secondary level of education while the lowest prevalence was found in women with tertiary level of education. This may be due to the fact that most of the participants had secondary level of education. Earlier reports found higher incidence in lower socioeconomic class [3,5,17] while majority of the women affected in this study were of the middle socioeconomic class and the least prevalence was in the high socioeconomic class. Improvement in the socioeconomic status of the participants may reduce the prevalence of AB amongst the study population. This study also showed higher prevalence in women aged 30 - 34 years and in primiparous women. Similar findings of higher prevalence among primiparous women have also been reported [1,3]. An earlier report of no association with parity and increased prevalence with lower maternal age by Hazhir was not corroborated by this study [17]. The exact link between parity, maternal age and AB is yet to be established. Klebsiella specie was the most prevalent organism isolated in 35.4% of cases followed by Escherichia coli in 24.6%. In contrast, most of the earlier reports showed Escherichia coli to be predominant organism in over 70% of the cases [15,19,20]. Only one study, by Akerele et al in Benin found similar distribution of microorganisms as found in this study [18]. Most (89%) of the organisms were sensitive to Nitrofurantoin. This high sensitivity to Nitrofurantoin has been reported by most of the earlier workers [8,20,21]. The study found no statistical difference in the mean Packed Cell Volume between the affected and unaffected women. Though 26% (17) of affected women were anaemic at booking compared to 24.5% (12) of unaffected women, this was not statistically significant, (p=0-963). This is in contrast to widely held view that asymptomatic bacteriuria is associated with anaemia in pregnancy [1,2]. Though there was no statistical difference in the incidence of urinary tract infection among the affected and unaffected women, women with AB were about 4 times more likely to develop UTI. This calls for increased surveillance for the detection of symptoms and signs of UTI among the study population and further study to determine the fetomaternal complications of UTI sequel to AB are recommended. The incidence of UTI of 10.8% among affected women is however lower than earlier reported incidences of about 30% [1,18]. This study did not demonstrate any statistically significant difference in pregnancy complications in women with AB when compared to women without AB even though women with AB were three times more likely to develop Pregnancy induced hypertension and puerperal sepsis. Other reports have associated asymptomatic bacteriuria with adverse pregnancy outcomes like preeclampasia, preterm labour, intrauterine growth restriction and prelabour rupture of fetal membranes [12,15,21]. Most of these reports in literature were not based on prospective comparative cohort studies of this nature. However, findings in this study are in support of findings from a multicenter prospective cohort study, which reported no significant adverse pregnancy outcome with AB [22]. There were no significant difference in the mean gestational age at delivery, mean birth weight and birth asphyxia between affected and unaffected women. This again is at variance with widely held view of low birth weight and preterm labour associated with AB [1,21]. A wider randomised control study and meta-analysis will help to resolve these conflicting reports in pregnancy outcome of AB.

## Conclusion

The prevalence of asymptomatic bacteriuria in pregnancy is high at UPTH. This prevalence is higher in primiparous and women of low socioeconomic status. There were no increased risk of adverse pregnancy outcomes such as anaemia, pre-eclampsia, intrauterine fetal death, low birth weight and birth asphyxia. Routine screening and treatment of AB is not recommended amongst the study population.

### What is known about this topic

- That asymptomatic bacteriuria occurs more in pregnancy due to the physiologic changes that occur in pregnancy;
- There are conflicting reports as to the association between asymptomatic bacteriuria and adverse pregnancy outcome;
- There are conflicting reports on whether asymptomatic bacteriuria in pregnancy should be treated or not.

### What this study adds

- This is the first study at the University of Port Harcourt teaching hospital to determine the impact of AB on pregnancy outcome;
- This study revealed a relatively high incidence of AB amongst the study population. It provides additional evidence on the association between AB and pregnancy outcome;
- That AB is not associated with adverse pregnancy outcome and thus negates the need for routine screening for AB amongst the study population.

## Competing interests

The authors declare no competing interest.
